# Three-dimensional classification of the Lenke 1 adolescent idiopathic scoliosis using coronal and lateral spinal radiographs

**DOI:** 10.1186/s12891-020-03798-x

**Published:** 2020-12-08

**Authors:** Saba Pasha, Victor Ho-Fung, Malcolm Eker, Sarah Nossov, Michael Francavilla

**Affiliations:** 1 Perelman School of Medicine, Department of Orthopedic Surgery, University of Pennsylvania, Philadelphia, PA USA; 2 Department of Radiology, University of Pennsylvania, Philadelphia, PA USA; 3 Department of Radiology, The Children’s Hospital of Philadelphia, Philadelphia, PA USA; 4 Department of Orthopedic Surgery, Shriners Hospitals for Children Philadelphia, Philadelphia, USA

**Keywords:** Adolescent idiopathic scoliosis, Classification, Three dimensional, Interobserver reliability, Intraobserver reliability

## Abstract

**Background:**

Classification of the spinal deformity in adolescent idiopathic scoliosis (AIS) remains two-dimensional (2D) as the spinal radiographs remain the mainstay in clinical evaluation of the disease. 3D classification systems are proposed, however are time consuming. We here aim to evaluate the clinical application of a 3D classification system by the use of only posterior-anterior and lateral radiographs in Lenke 1 adolescent idiopathic scoliosis (AIS).

**Methods:**

Forty Lenke 1 AIS were classified by five observers following a three-step flowchart, developed based on our previous 3D classification system. This 3D classification characterizes the curve in the frontal and sagittal views and infers the third dimension with rules based on prior data to determine the 3D subtypes of the curve. Repeated rating was performed for 20 randomly selected patients in the same cohort. In addition to the classification by the raters, the 3D model of the spines were generated to determine the actual curve subtype based on the algorithm that was originally used to develop the 3D classification system. The interobserver and intraobserver reliability and the classification accuracy were determined for both 3D and axial classifications of the cohort.

**Results:**

The interobserver reliability was moderate to strong with a kappa value between 0.61–0.89 for 3D and axial classifications. Comparing the mathematical classification and the raters’ classification, the classification accuracy among all raters ranged between 56 and 89%.

**Conclusion:**

We evaluated the reliability of a previously developed 3D classification system for Lenke 1 AIS patients when only two-view spinal radiographs are available. Radiologists and orthopedic surgeons were able to identify the 3D subtypes of Lenke 1 AIS from the patients’ radiographs with moderate to strong reliability. The new 3D classification has the potential to identify the subtypes of the Lenke 1 AIS without a need for quantitative 3D image post-processing.

## Introduction

Classification systems of spinal deformities in adolescent idiopathic scoliosis (AIS) have been proposed to guide surgical decision-making [1, 2]. Two dimensional (2D) spinal radiographs remain the mainstay of the clinical evaluation in AIS, thus the classifications remain based on the sagittal and coronal characteristics of the curves. Yet, scoliosis is known to be a three dimensional deformity. Several studies have shown that the three dimensional (3D) characteristics of the curve play an important role in surgical decision-making and surgical outcomes prediction [3–6].

The 3D characteristics of the scoliotic curves, using mathematical and statistical methods, have been evaluated [4, 7]. Such quantitative analyses have identified different 3D curve patterns in AIS Lenke subtypes [4, 7]. However, the additional sophisticated image post-processing and mathematical analysis that are required to identify these different 3D variants have hampered clinical application of such methods.

Previously, we developed a statistical 3D classification of right thoracic AIS using 3D models of the spine [4]. Subsequently, we identified characteristics of these subtypes in the coronal and sagittal views (posterior-anterior (PA) and lateral radiographs) that could be identified on the 2D spinal radiographs to infer the axial view. As this classification proved to provide meaningful clinical guidelines for surgical planning and surgical outcome risk stratification [5], we aimed to assess the reliability of identifying these 3D subtypes using two-view 2D radiographs (PA and lateral) of the spine.

To this end, we evaluated the inter- and intra-observer reliability of our 3D classification system of Lenke1 AIS using the 2D characteristics of the curves. We particularly focused on this subtype of scoliosis as identifying the 3D subtypes is shown to be beneficial in selecting the fusion levels [5]. It was hypothesized that trained raters can reliably identify the 3D subtypes of the right thoracic AIS using the descriptive characteristics of the curve determined on the 2D radiographs.

## Methods

### Cohort specifications

After obtaining institutional review board approval, we retrospectively obtained a sample of a total of 40 consecutive pre-surgical Lenke 1AIS patients, 12–17 years old. All patients had two view orthogonal calibrated stereoradiography images (EOS imaging, Paris, France)- posterior-anterior and lateral spinal radiographs. The bender films were used to determine lumbar curve flexibility. We included male and female patients with a main thoracic Cobb angle greater than 45**°** and lumbar Cobb angle that corrects to below 25**°** on the bending films. Per Scoliosis Research Society definition of the right thoracic curve, the apex of thoracic curve was above the T11-T12 intervertebral disc. Patients with prior spinal or hip surgery, neuromuscular conditions, spondylosis and spondylolisthesis, vertebrae congenital abnormalities including supernumerary were excluded.

### 3D Scoliotic subtypes characteristics

**Fig. 1:**
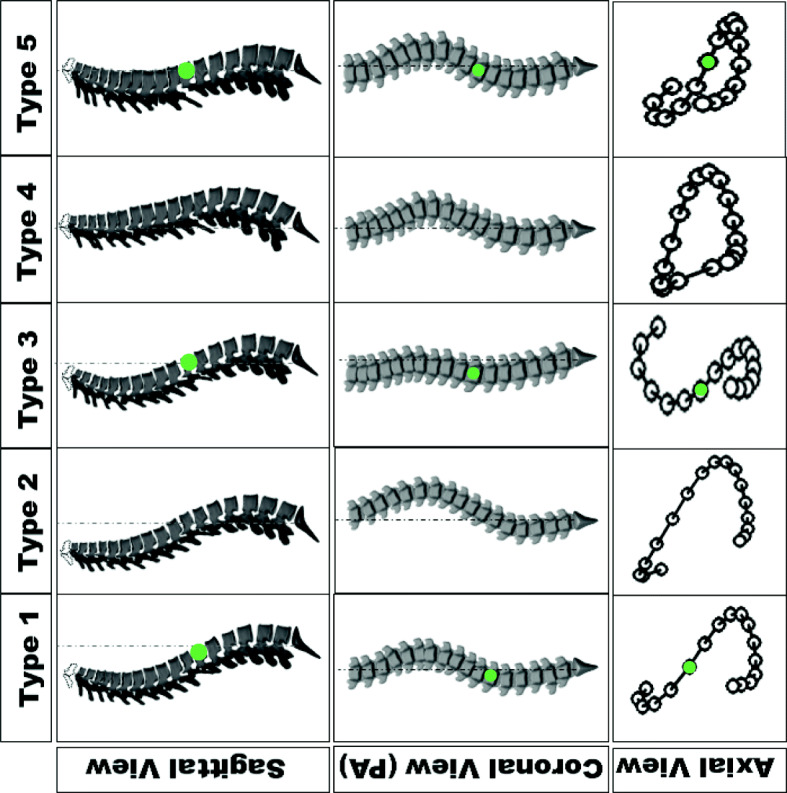
Five subtypes of Lenke1 AIS. The spinal curvatures are shown in the sagittal, frontal (posterior-anterior), and axial (Top-down) views. The vertebra at which the direction of the vertebral location changes in the thoracolumbar section is shown with a green circle

The spinal characteristics of the scoliotic subtypes in the PA and sagittal views were determined from a previous study [4]. This study described five subtypes in a cohort of right thoracic AIS by determining a series of qualitative specifications of the spinal curves in the coronal, sagittal, and axial (determined from the pattern of the pedicles’ orientation in the PA radiographs) views (Fig. 1). The spinal curve patterns in these 5 subtypes are:

- **Type 1:** *Sagittal view*: hyperkyphotic/normal [2] sagittal profile and negative sagittal vertical axis (SVA)- C7 behind the posterior aspect of the sacrum. The kyphotic section of the spine is longer than the lordotic section of the spine, i.e., the kyphosis was extended to thoracolumbar spine. *PA view*: A compensatory curve above the main thoracic curve is observed thus T1 is leveled or tilted to the right side (in posterior view). *Axial view*: The direction of the vertebral rotation changes in the thoracolumbar region. The axial projection of the curve appears as lemniscate (figure-eight) shape.
- **Type 2**: *Sagittal view*: Hypokyphotic [2] with no proximal kyphosis and negative SVA. The lordotic section is longer than the kyphotic section of the spine, i.e.*,* the thoracolumbar section is lordotic. *PA view*: no proximal curve above the main thoracic curve thus T1 is leveled or tilted to the left in posterir view. *Axial view*: The direction of the vertebral rotation changes in the lower lumbar region or does not change at all below the caudal neutral vertebra – the neutral vertebra located below the main thoracic apex- The axial projection of the curve appears as loop shaped.
- **Type 3:** *Sagittal view*: hypokyphotic at T5-T10 levels, close to zero SVA. *PA view*: frontal imbalance and trunk shift, T1 is leveled or tilted to the right due to a compensatory curve in proximal thoracic. *Axial view*: The direction of the vertebral rotation changes in the thoracolumbar region (lemniscate axial view).
- **Type 4**: *Sagittal view:* Flat (no substantial kyphosis/lordosis) or lordotic sagittal profile with positive SVA, high (above midpoint of the spinal curve) sagittal inflection point thus the lordotic section of the spine is longer than the kyphotic section. *PA view*: no proximal thoracic curve, T1 is leveled or tilted to the left as the main thoracic curve continues to the proximal thoracic. *Axial view*: The vertebral rotation changes direction in the lower lumbar region or does not rotate below the caudal neutral vertebra (loop shaped axial view).
- **Type 5**: *Sagittal view:* hypokyphotic and forward trunk shift, positive SVA with low inflection point (below midpoint of the spine) and a proximal kyphosis curve. *PA view*: T1 is leveled or tilted to the right. *Axial view*: the direction of the vertebral rotation changes in the thoracolumbar region (lemniscate axial view).

### Manual classification

**Fig. 2:**
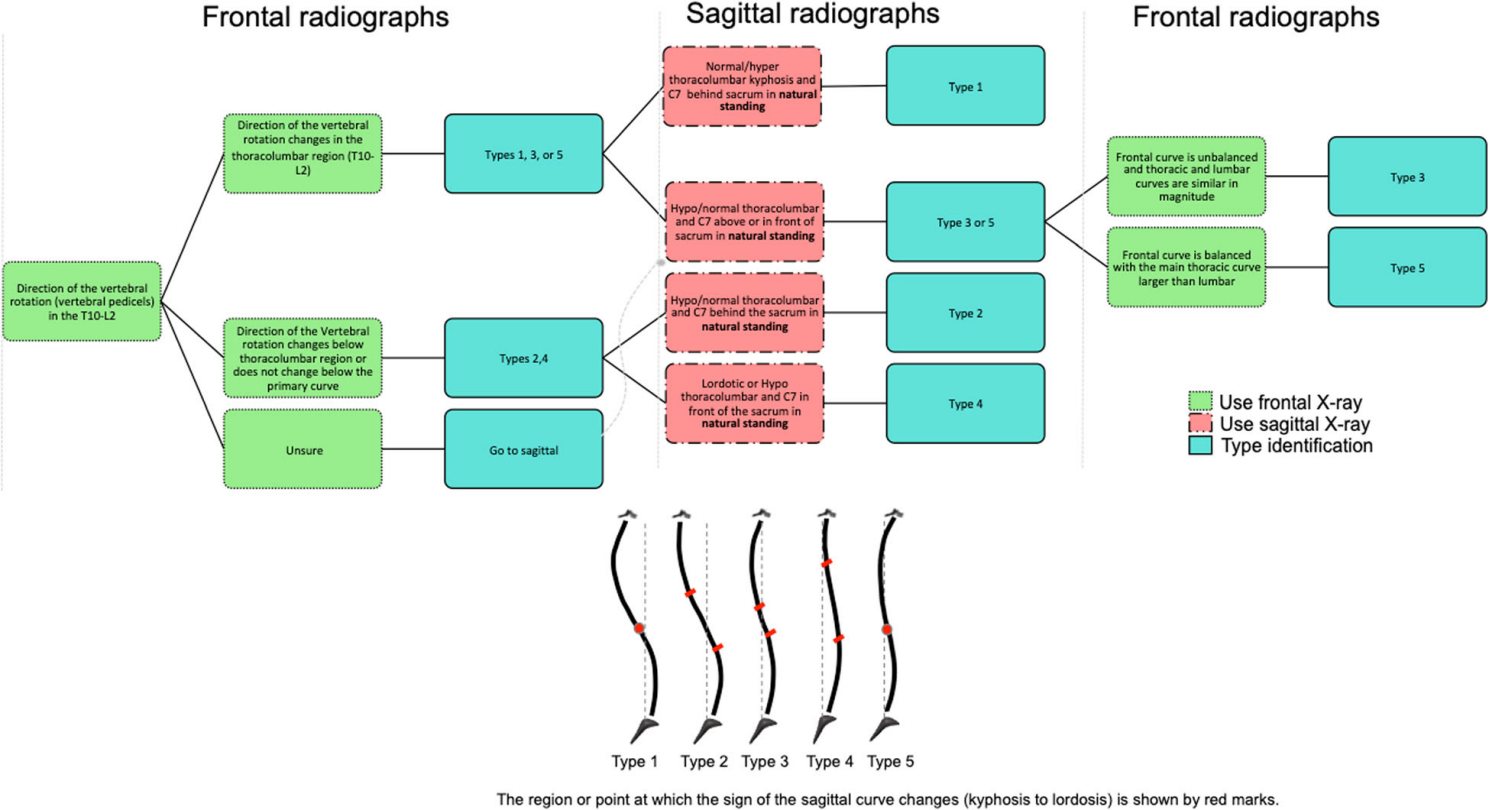
**a** Three steps classification flowchart, using the 2D radiographs, is shown. The raters used frontal, sagittal and again frontal radiographs (when case 3 or 5 were selected) to determine the 3D subtypes. **b** Schematics of the sagittal views. The length of the spine over which the sign of the sagittal curve changes (kyphosis to lordosis) is shown by two small lines

Figure 2 shows the three steps that were used in manual classification by the five raters. A flowchart was created based on the curves characteristics in the three anatomical planes to guide the raters through the classification process (Fig. 2a). The schematic of the sagittal profiles that shows the kyphotic/lordotic sections of the spine and the position of the inflection point or the section of the spine without a curve (inflection region) are shown in Fig. 2b.

### Raters and classification process

A total of five raters, two pediatric orthopedic spine surgeons, two pediatric radiologists, and one research engineer performed the classification. To assess interobserver reliability, the four physicians repeated the classification for a subset of randomly selected 20 patients in the same cohort. Raters were trained in person to use the flowchart (Fig. 2). Raters accessed the medical images independently and recorded their rating in Research Electronic Data Capture (REDCap). Raters also determined the kyphosis and lumbar modifiers based on the Lenke classification for each patient [2].

### Image processing

The 3D reconstructions of the spines were generated in SterEOS 2D/3D software (EOS imaging, Paris, France) for all the 40 subjects. The vertebral bodies centroids were used to generate the spinal centerline using a previously described method [8]. In addition, the 3D spinal centerlines of the five subtypes, determined in a previous study, were included [4]. These five 3D curves (spinal centerlines) were used to determine the similarity between the new cases (each of the 40 patients) to one of the original five 3D curves, as shown in Fig. 1.

### Statistical analysis

To determine the subtype of the new data points (40 patients) we created a dissimilarity matrix between the five original subtypes of 3D spinal curves (Fig. 1) and each of the 40 new patients by calculating the Euclidean distances between each pair. The dissimilarity matrix, as the basis of many clustering methods, determines a quantitative measure of dissimilarity between the cluster centers and each new patients and determines to which of the existing clusters the new patient belongs. As such, the dissimilarity between the subjects within each cluster is significantly smaller than the dissimilarity between the subjects from two different clusters. The smallest dissimilarity between the patients’ spinal 3D curve and the existing 5 cluster’s centerlines determined the new patients’ subtype (Types 1 to 5). If the dissimilarity between the new patient’ 3D spinal curve and the five clusters were larger than the dissimilarity between the five clusters, that patient was excluded from the inter- and intra-observer reliability analysis.

The inter- and intra-observer reliabilities were evaluated between and within the raters using Fleiss’s Kappa. The rating reliabilities were evaluated separately for the 3D classification (identifying subtypes 1 to 5) and the axial classification (loop and lemniscate subtypes) (Fig. 1- axial view). We also calculated the accuracy of the visual ratings by each observer, considering the numerical classification as the true observation (gold standard), using the number of true positive classifications in the confusion matrix.

## Results

**Fig. 3:**
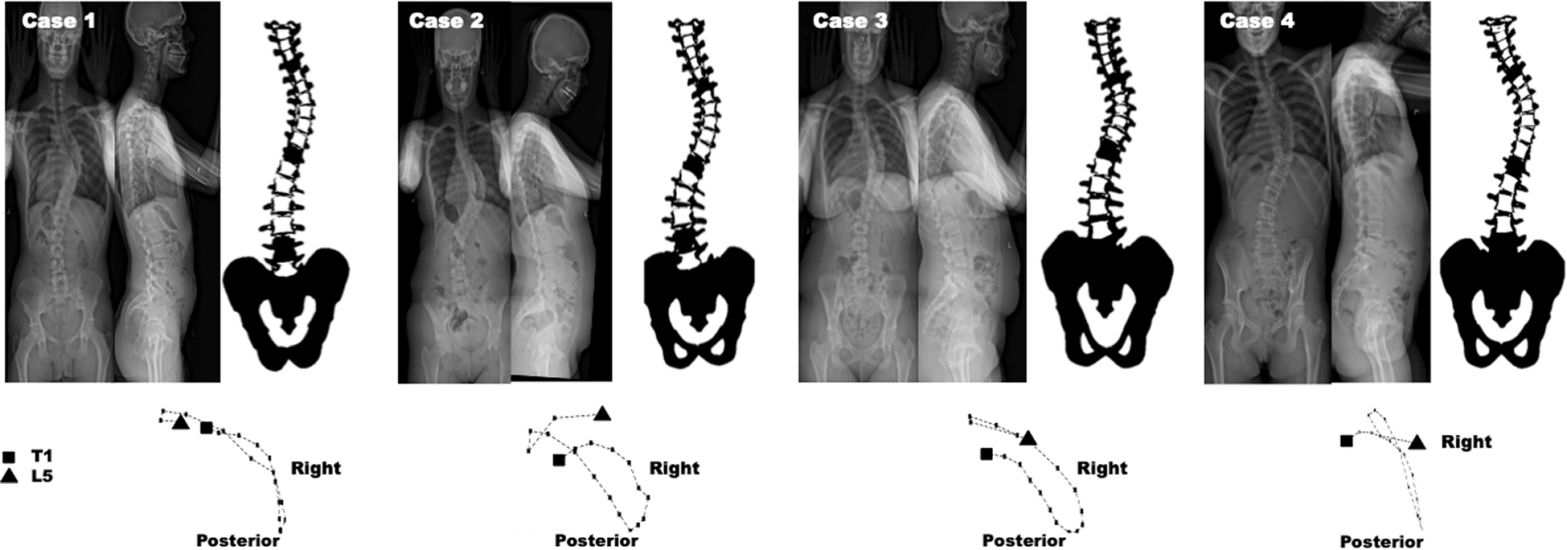
Presentation of four cases with a large dissimilarity to the five subtypes of Lenke 1 (per our previous classification). The PA and lateral radiographs, the axial projection, and the 3D model of the spine are shown. The end vertebrae are colored in the 3D images. The position of the T1 and L5 vertebrae in the axial is shown

A total of 4 out of 40 patients were excluded from the analysis based on the distances calculated in the dissimilarity matrices (Table 1). The dissimilarity between the 3D spinal curves of these patients and all the five existing cluster centers was larger than the between cluster dissimilarities (Table 1). Table 1 shows the dissimilarity matrix for the five clusters and these four cases. Figure 3 shows the PA and lateral spinal radiographs of these cases. Only one of these patients was in the cohort that was selected randomly for interobserver reliability assessment. Table 2 summarizes the average and standard deviation of the frontal Cobb angles and sagittal parameters of the remaining 36 patients in the 5 subtypes based on the numerical (true) clustering.

**Table 1:** The cluster assignment based on the dissimilarity matrix. The minimum Euclidian distance for each patient, determined the type. For example *patient 1* was assigned to Type 1 as the calculated Euclidean distance between the Type 1 and Patient 1 was smaller than the distance between the patient 1 and other Types. Four patients were excluded as the dissimilarity between these cases and the five types exceeded the Euclidian distance between the five types as shown in the shaded section of the table

Patient ID	Type 1	Type 2	Type 3	Type 4	Type 5	
Type 1	0					
Type 2	444.5	0				
Type 3	549.5	350.9	0			
Type 4	365.6	279.3	312.7	0		
Type 5	514.3	572.2	448.7	301.7	0	
Patients ID	Type 1	Type 2	Type 3	Type 4	Type 5	Assigned cluster
1	318.3	632.9	630.8	596.7	930.6	1
2	422.8	221.5	690.5	425.1	557.2	2
3	614.6	336.8	617.8	379.8	212.9	5
4	718.8	576.2	545.7	476.1	236.4	5
5	960.2	825.0	658.1	675.5	393.0	5
6	150.1	321.5	557.5	372.3	673.7	1
7	300.2	238.9	357.7	83.1	382.1	4
8	225.4	609.2	425.4	395.7	628.2	1
9	278.6	406.5	238.0	205.0	487.3	4
10	916.5	868.2	717.8	980.9	700.9	X
11	549.2	392.6	431.9	287.0	180.8	5
12	180.5	422.9	386.2	323.1	628.7	1
13	282.5	306.8	656.1	422.5	704.5	1
14	636.4	808.0	537.8	263.1	730.8	4
15	854.5	884.2	790.3	905.7	889.7	X
16	693.5	678.0	432.6	339.6	478.0	4
17	348.2	270.6	445.7	215.8	421.5	4
18	575.1	700.1	239.9	426.1	188.6	5
19	511.2	211.2	656.7	366.4	445.4	2
20	287.0	210.2	396.1	136.3	421.0	4
21	431.4	630.9	150.8	386.9	627.4	3
22	675.8	392.2	644.7	427.7	226.9	5
23	952.1	590.6	778.2	1143.1	693.5	X
24	219.0	337.1	551.8	364.1	658.5	1
25	388.5	508.5	227.6	262.8	545.2	3
26	278.1	608.6	490.6	513.7	825.3	1
27	407.9	167.1	659.6	392.8	512.9	2
28	235.8	559.6	454.4	463.0	775.7	1
29	311.7	166.8	544.8	292.2	468.0	2
30	680.3	756.2	734.2	811.4	703.1	X
31	262.9	190.3	601.5	360.4	582.3	2
32	331.0	117.2	572.0	307.1	478.1	2
33	336.9	280.3	428.2	204.9	426.9	4
34	283.3	204.8	385.6	103.6	403.3	4
35	327.1	215.5	440.6	164.1	429.7	4
36	495.6	471.4	255.7	230.2	339.9	4
37	333.3	220.4	536.5	328.8	591.9	2
38	446.7	550.4	121.3	277.4	510.3	3
39	370.6	361.5	232.7	110.4	341.9	4
40	532.8	622.5	143.1	360.8	511.6	3

**Table 2:** Patients’ clinical parameters. The type specification was based on the numerical class assignment using the dissimilarity matrices

	Thoracic Cobb angle (°)	Lumbar Cobb angle (°)	T5-T12 Kyphosis (°)	L1-S1 Lordosis (°)	Sacral slope (°)	Pelvic incidence (°)
Type 1, *n* = 8	51 ± 5	38 ± 11	24 ± 7	55 ± 9	43 ± 6	49 ± 8
Type 2, *n* = 7	55 ± 8	32 ± 5	18 ± 8	51 ± 7	44 ± 8	52 ± 7
Type 3, *n* = 4	46 ± 7	42 ± 6	20 ± 8	51 ± 8	44 ± 6	48 ± 8
Type 4, *n* = 11	54 ± 8	33 ± 4	13 ± 7	53 ± 7	43 ± 10	53 ± 9
Type 5, *n* = 6	52 ± 4	35 ± 6	17 ± 5	54 ± 7	42 ± 7	50 ± 8
All patients, *n* = 36	52 ± 6	35 ± 7	18 ± 7	52 ± 7	43 ± 7	50 ± 8

For the 3D classification of the curves, the interobserver reliability (for 19 patients and 4 observers) was moderate [9], κ = 0.69. The intraobserver reliability for 3D classification (for 36 patients and 5 observers) was moderate, κ = 0.61. The intraobserver reliability for identifying kyphosis modifier was moderate, κ = 0.78 and for lumbar modifier was strong, κ = 0.86.

For the axial classification, the interobserver reliability was almost perfect, κ = 0.91 and the intraobserver reliability was strong, κ = 0.80.

The balanced accuracy value (the proportion of the correct prediction for each subtype) for raters 1 to 5 are shown in Table 3. The classification accuracy was the lowest for Type 2, 0.63, 95% CI = [0.57,0.67] and the highest accuracy for Types 4, 0.76, 95% CI = [0.73,0.79] among all raters.

**Table 3:** Classification accuracy (number of correct observations divided by the total number of observations) of the five raters and for each subtype

	Type1	Type2	Type3	Type4	Type5	Overall per rater, [95%CI]
Rater1	0.75	0.72	0.50	0.82	0.83	0.75, [0.71, 0.79]
Rater2	0.88	0.86	0.75	0.90	1.00	0.89, [0.86, 0.92]
Rater3	0.63	0.57	1.00	0.72	0.33	0.58, [0.51, 0.66]
Rater4	0.50	0.42	0.50	0.72	0.50	0.56, [0.52, 0.59]
Rater5	0.75	0.57	0.50	0.64	0.66	0.64, [0.61, 0.67]
Overall per type, [95%CI]	**0.70, [0.65,0.74]**	**0.62, [0.57,0.67]**	**0.65, [0.58,0.72]**	**0.76, [0.73,0.79]**	**0.67, [0.58,0.75]**	

## Discussion

Two-dimensional classification systems of the scoliotic spine are developed to assist with surgical planning [2, 10, 11]. Yet, variations in the surgical outcomes exist partially due to the fact that the 3D shapes of the scoliotic spines are not incorporated in these classification systems [12–16]. We developed a true 3D classification of the right thoracic scoliosis and attempted to apply the classification system via 2D images to identify the characteristics of these 3D subtypes on 2D radiographs. This study determined the reliability of this 3D classification method based on 2D radiographs to be moderate to strong among the raters suggesting that this 3D classification system has the potential to be used in orthopedic clinics without a need for excessive image post-processing.

The reliability of the 3D classification system proved to be within the range of the Lenke modifier kappa values in the cohort of our raters and superior to other classification system commonly used in the field of orthopedics. For example the kappa value for tibial plateau fractures classifications was 0.476 based on Schatzker classification [17]. Similarly the kappa for pediatric supracondylar fractures is reported at 0.475 for Wilkins-modified Gartland classification [18]. Our intraobserver reliability for the 3D classification and axial classification were at κ = 0.61 and 0.80 respectively suggesting an acceptable range for clinical applications. The intraobsever reliability varied among the five raters; however as the number of raters is small we did not make any relationship between the raters (surgeon versus radiologist, versus engineer) and the reliability scores.

Surgical planning in AIS aims to stabilize the spine while minimizing the number of the fused vertebrae [19, 20]. In doing that, short fusion or variations in the upper and lower instrumented vertebrae (UIV and LIV) may result in compensatory curve progression, postural compensation, and subsequently a need for revision surgery [5, 20]. Our classification primarily focuses on identifying the true 3D characteristics of these curves by utilizing axial information to augment our understanding of the curve in right thoracic AIS [4, 21]. Our previous results demonstrated that the vertebral level below the thoracic apex at which the direction of the vertebral rotation changes can identify the true number of the 3D curves and the axial subtypes in right thoracic AIS [4, 21]. The lemniscate axial type (with a rather sudden change in the direction of the vertebral rotation in the thoracolumbar region) responds better to shorter fusion whereas loop shaped axial types (with a long sweeping curve and change in the direction of the vertebral rotation in the lower lumbar region or no rotation at all below the apex) require an extension of the fusion to the lumbar spine [5] as the spine is comprised of only one long 3D curve. Yet, detailed surgical planning based on this classification remains to be further established. The criterion based on the vertebral rotation is close to the concept of the neutral vertebra (NV), however current definition tries to draw attention to whether the least rotated vertebra is located between two curves (if the vertebral rotates to the opposite direction below NV i.e., lemniscate axial group) or part of the structural curve (if the vertebral rotation does not change below the NV, i.e.*,* loop shaped axial group) [5]. A previous risk stratification analysis based on the 3D classification that was presented here showed that the subtypes with loop shape axial projection benefit from longer fusion that includes the entire 3D curve whereas fusion of one of the 3D curves in subtypes with lemniscate shaped axial projection can improve the rate of spontaneous lumbar Cobb angle correction [5]. The raters in our study could identify the axial subtypes with excellent reliability only by considering the pattern of the vertebrae rotation in the thoracolumbar/lumbar sections. This axial classification of the right thoracic AIS, as described in this study, can provide a better understanding of the nature of the compensatory curves in right thoracic AIS and assist with surgical decision-making.

As our 3D classification includes the sagittal alignment of the spine, attention should be paid to natural patient positioning during radiograph acquisition [22, 23]. As the importance of sagittal alignment in clinical evaluation of scoliosis is emphasized [21, 24, 25], patient positioning methods that do not change the postural alignment are critical for sagittal evaluation of the spine. Considering the sagittal profile in natural standing position, Types 1 and 2 have a negative SVA thus excessive transition of the UIV anteriorly, while imparting kyphosis, can result in proximal junctional kyphosis due to over-working of the posterior elements [5, 26]. Types 4 and 5 have a positive SVA. Imparting large kyphosis may result in disturbing the harmonious spino-pelvic alignment and developing compensatory mechanism [26]. Finally, the changes in the position of UIV are of greater importance in the curve types without a proximal kyphosis i.e., Types 2 and 4 as shown in a previous analysis [5]. This shows the importance of the proximal kyphosis which was not considered in previous sagittal classification of the spine [27, 28].

All raters primarily used the flowchart to perform the classifications (Fig. 2a). A total of 4 subjects were excluded from the current analysis because a large dissimilarity between the 3D spinal curvature of these patients and the original cluster centers as determined in our previous work [4, 5] was observed (Fig. 3 and Table 1). When visually evaluated, these patients had one or more characteristics that did not fit in the 3D description of any of the clusters (Figs. 1 and 2). For example, case 1 presented with a hypokyphosis and flat sagittal profile, as seen in Type 4, but with slight rotation of the thoracolumbar curve to the opposite direction of the thoracic curve, similar to what is seen in Types 1, 3, and 5 (Fig. 3-a). Case 2 had a sagittal profile similar to Type2 but slight vertebral rotation in the thoracolumbar region (Types 1, 3, or 5) was observed. Case 3 had a sagittal profile similar to Type 3 or 5 however no vertebral rotation in the thoracolumbar region was observed, suggesting a Type 2 or 4. Finally, case 4 had Type 4’s sagittal profile but the frontal curve, based on the vertebral rotation below the neutral vertebra, was classified as Types 1, 3, or 5. Analysis of a larger database can determine whether these cases should be considered as additional subgroups of Lenke1 AIS. Extra attntion needs to be paid during the radiograph acquisation and avoid patient positionings that alter the natural sagittal profile [23].

Advancements in deep learning in medical imaging can further facilitate this classification method. Such automated methods can determine new subgroups if the similarity between the patients’ subtypes increased and form new groups using an online processing algorithm. This is the subject of our future work. Using this 3D classifications algorithm [29] while including true 3D parameters of the spine [30], method needs to be expanded to other Lenke types and validated. Finally, an external validation of this classification is required to show whether this classification system can be used reliably in other hospitals and research centers.

## Conclusion

Although the 3D reconstruction of the spine can contribute significantly to our understanding of the scoliosis spine, the proposed rule-based classification using 2D radiographs can provide valuable and readily accessible information to orthopedic clinics using the technology already utilized in the clinical setting.

## Data Availability

The patients’ dataset are confidential and are privately held for patients confidentiality safeguard. As such, the datasets generated and/or analysed during the current study are not publicly available but are available from the corresponding author on reasonable request.

## Abbreviations

2D: Two-dimensional
3D: Three-dimensional
AIS: Adolescent idiopathic scoliosis
CI: Confidence interval
LIV: Lower instrumented Vertebra
NV: Neutral Vertebra
PA: Posterior-anterior
REDCap: Research Electronic Data
SVA: Sagittal vertical axis
UIV: Upper instrumented vertebra
